# Angioplasty With Stent Implantation for Portal Venous Stenosis Caused by Abdominal Tuberculosis: A Case Report and Literature Review

**DOI:** 10.3389/fmed.2021.778672

**Published:** 2021-10-28

**Authors:** Xin Quan, Yang Tai, Bo Wei, Huan Tong, Zhidong Wang, Yuhang Yang, Hao Wu

**Affiliations:** ^1^Department of Gastroenterology, West China Hospital, Sichuan University, Chengdu, China; ^2^Laboratory of Gastroenterology and Hepatology, West China Hospital, Sichuan University, Chengdu, China

**Keywords:** case report, abdominal tuberculosis, portal venous stenosis, portal hypertension, portal venous angioplasty

## Abstract

Abdominal tuberculosis is one of common forms of extra-pulmonary tuberculosis. However, portal vein involvement leading to portal venous stenosis and portal hypertension is a rare complication in abdominal tuberculosis. Because of the non-specific presentations and insensitive response to anti-tuberculosis therapy of the lesions involving portal vein, it continues to be both a diagnostic and treatment challenge. We have reported a 22-year-old woman presented with massive ascites and pleural effusion, which was proved to be TB infection by pleural biopsy. After standard anti-tuberculosis therapy, her systemic symptoms completely resolved while ascites worsened with serum-ascites albumin gradient >11 g/L. Contrast-enhanced computed tomography and portal venography showed severe main portal vein stenosis from compression by multiple calcified hilar lymph nodes. Finally, the patient was diagnosed with portal venous stenosis due to lymphadenopathy after abdominal tuberculosis infection. Portal venous angioplasty by balloon dilation with stent implantation was performed and continued anti-tuberculosis therapy were administrated after discharge. The ascites resolved promptly with no recurrence occurred during the six-month follow-up. Refractory ascites due to portal venous stenosis is an uncommon vascular complication of abdominal tuberculosis. Portal venous angioplasty with stent placement could be a safe and effective treatment for irreversible vascular lesions after anti-tuberculosis therapy.

## Introduction

Abdominal tuberculosis (TB) is one of the most common forms of extra-pulmonary TB, which represents about 10–12% of all extra-pulmonary TB infection (1, 2). The infection of *Mycobacterium tuberculosis* (*M. tuberculosis*) can involve any intra-abdominal organ and peritoneum, resulting in protean clinical manifestations (3). Although portal vein involvement is rare at initial presentation of abdominal TB, vascular complications may occur due to the direct invasion of the vascular wall by *M. tuberculosis* or regional constriction of tuberculous masses (4). At present, standard anti-tuberculosis therapy (ATT) is effective for abdominal TB, however, vascular complications remain a therapeutic challenge because of the insensitive response to ATT (5). Herein, we presented a case with portal venous stenosis and portal hypertension (PHT) caused by abdominal TB, and an interventional strategy for irreversible vascular lesions.

## Case Presentation

A 22-year-old woman presented with abdominal distention and intermittent left lower quadrant pain for 3 weeks. She also complained of hot flashes, night sweat, anorexia, and weight loss of 5 Kg within 20 days. Laboratory examination in the primary hospital revealed the serum-ascites albumin gradient (SAAG) <11 g/L (albumin level in serum and ascites were 34.3 g/L and 24.2 g/L, respectively), but no malignant cells was found in ascitic fluid specimen. The diuretics had limited therapeutic effects on relieving symptoms, and the patient was transferred to our hospital for further diagnosis and treatment thereafter.

**Figure 1:**
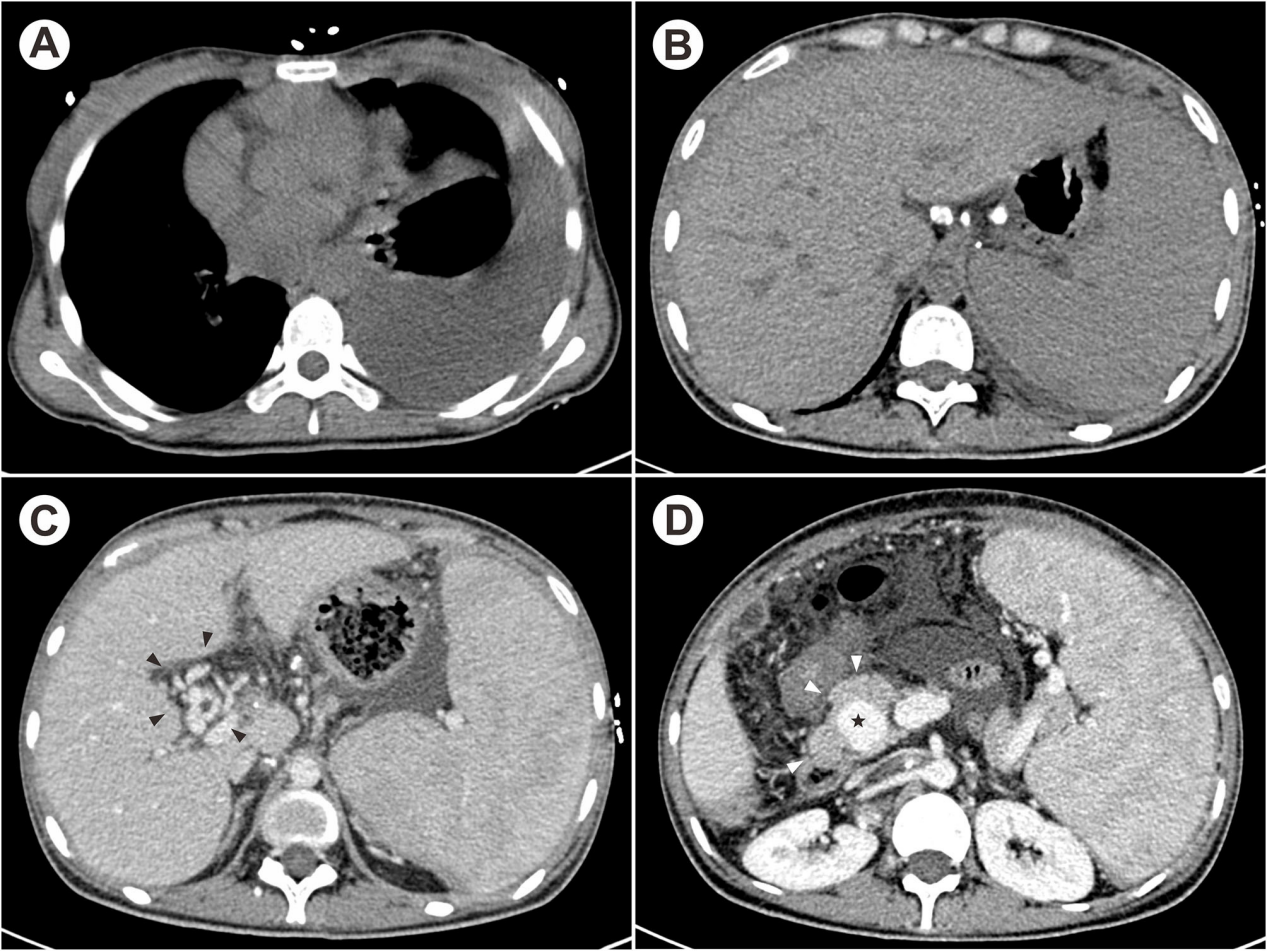
The contrast-enhanced CT showed massive pleural effusion on the left chest **(A)**, multiple calcified hilar lymph nodes **(B)**, CTPV [**(C)**, black arrowheads] and distal portal venous aneurysm [**(D)**, star] due to lymphadenopathy [**(D)**, white arrowheads].

On admission, physical examination was significant for pale skin, percussive dullness on the left chest and left lower quadrant tenderness with shifting dullness. Laboratory tests showed the decreased hemoglobin (80 g/L, normal 115–150 g/L), decreased leukocyte (2.17 × 10^9^ /L, normal 3.5–9.5 × 10^9^ /L), normal platelet counts, increased aspartate aminotransferase (53 IU/L, normal <35 IU/L), decreased albumin (35 g/L, normal 40–55 g/L), and prolonged prothrombin time (14.5 s, normal 9.6–12.8 s). The erythrocyte sedimentation rate (ESR) and C-reactive protein (CRP) were both elevated (45 mm/h and 95 mg/L, respectively). Severe esophageal varies was found by esophagogastroduodenoscopy. Contrast-enhanced computed tomography (CT) suggested massive pleural effusion on the left chest (Figure 1A), swollen and thickening peritoneum, and multiple enlarged and calcified lymph nodes located at porta hepatis and peripancreatic regions (Figure 1B). The portal venous stenosis resulted in cavernous transformation of portal vein (CTPV), distal portal venous aneurysm, and enlarged spleen with dilated splenic vein (Figures 1C,D). Additionally, pleural fluid proves to be exudative with an elevated adenosine deaminase (ADA) level (41 IU/L) and ultrasound-guided pleural biopsy was performed afterwards. The histopathological changes showed granulomatous inflammation and TB-DNA was detected by real-time quantitative polymerase chain reaction. Conclusively, the patient was diagnosed with extrapulmonary TB involved the pleura, peritoneum and celiac lymph nodes.

**Figure 2:**
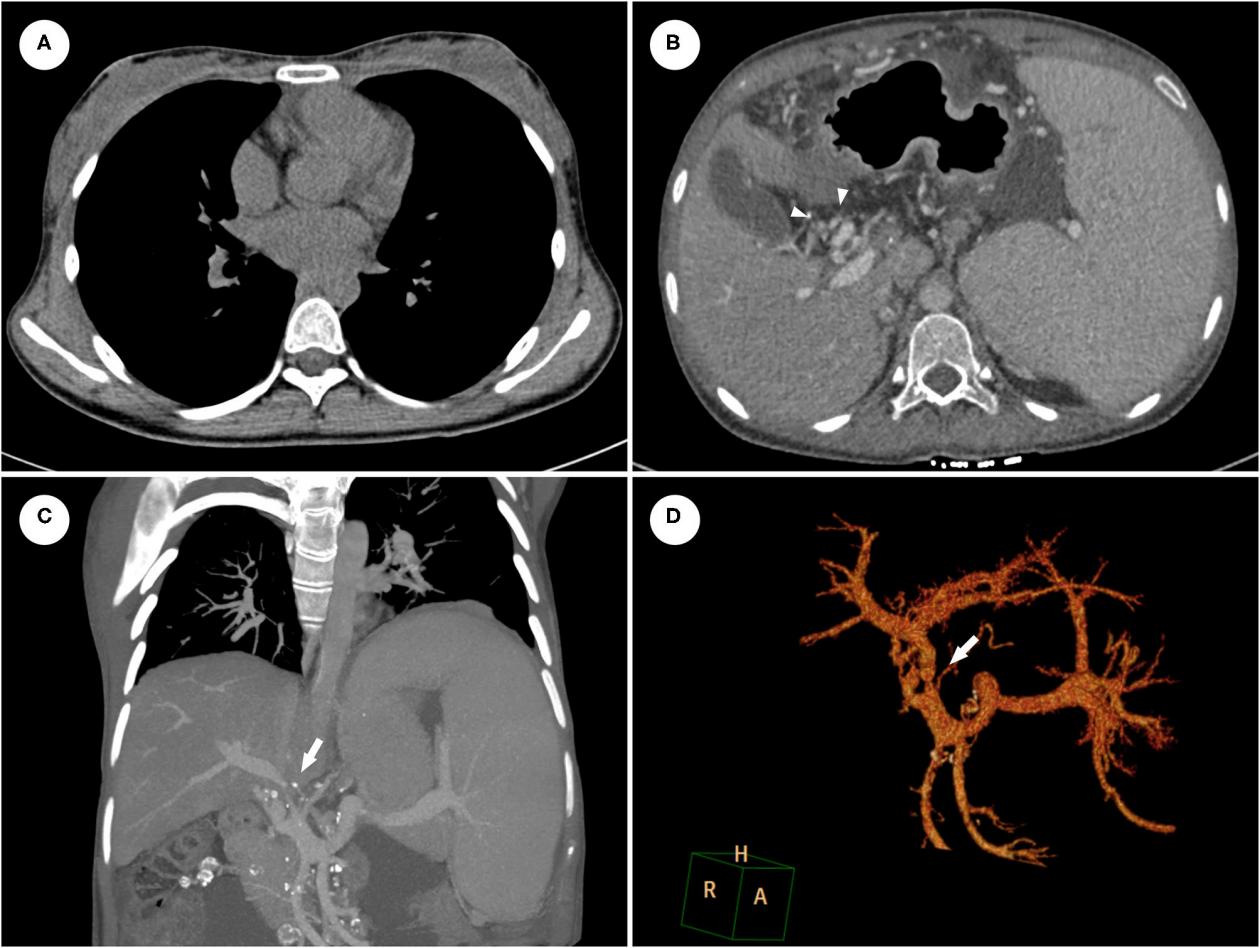
The contrast-enhanced CT revealed that pleural effusion disappeared **(A)** but CTPV [**(B)**, arrowheads] was not improved after ATT. CTA with three-dimensional reconstruction showed severe stenosis of main portal vein **(C,D)**.

**Figure 3:**
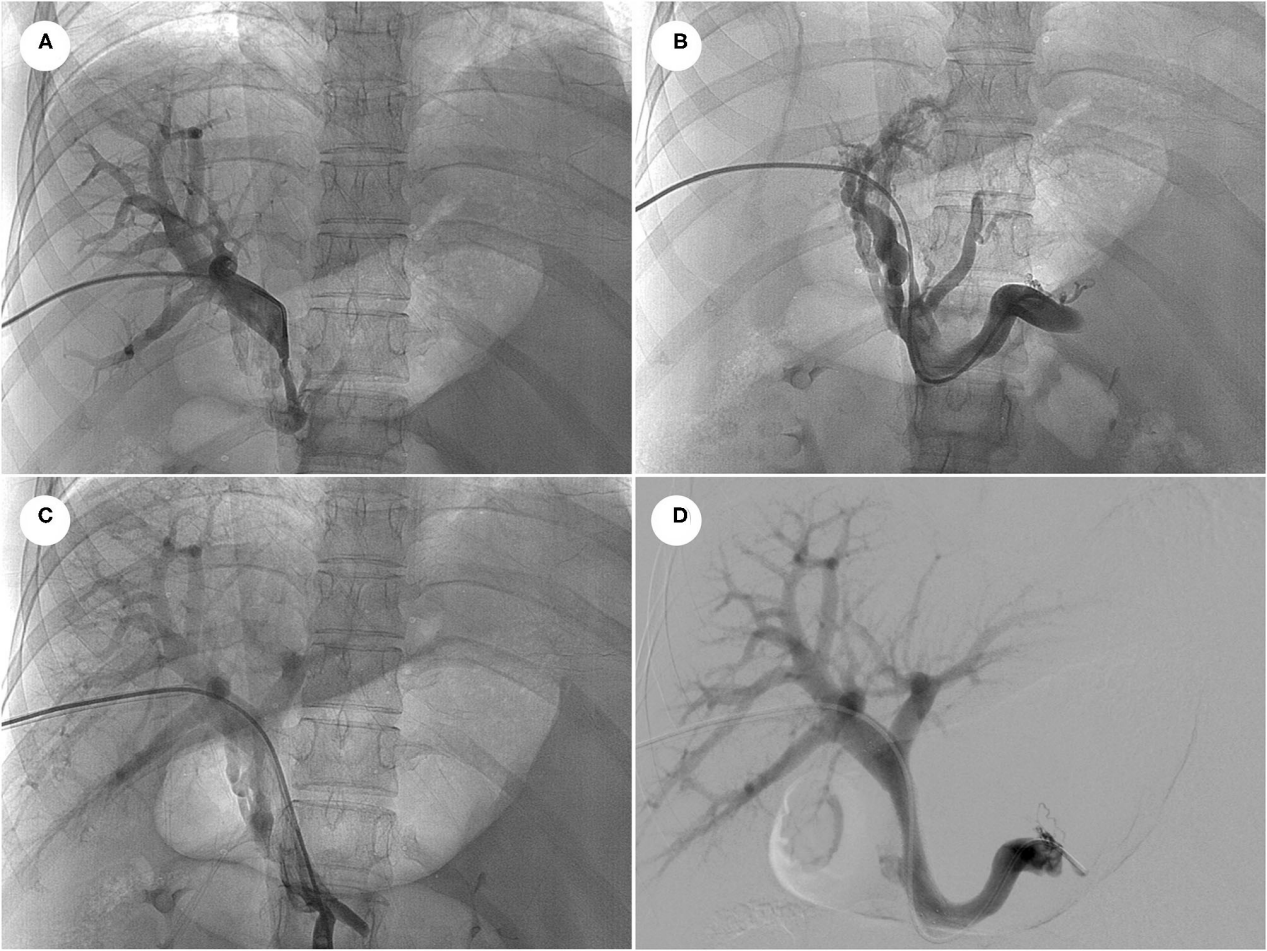
Portal venography showed the main portal vein underwent severely stenosis to be nearly occlusion at the hepatic hilum **(A)**, and distal portal vein distributed abundant varices to encompass the hepatic hilum **(B)**. After portal venous angioplasty by balloon dilation **(C)** and stent implantation **(D)**, portal vein at the hepatic hilum was clearly displayed, and collateral varices disappeared.

The symptoms completely resolved after six-month ATT with the combination of isoniazid, rifampicin, ethambutol and levofloxacin. ESR and CRP returned to the normal range and chest CT revealed the effective control of pleural effusion (Figure 2A). However, the abdominal distension with massive ascites recurred six months later. The SAAG (>11 g/L, albumin level in serum and ascites were 42.4 g/L and 28.2 g/L, respectively) indicated the ascites is due to PHT. Furthermore, abdominal CT angiography (CTA) with three-dimensional reconstruction showed severe stenosis of main portal vein constricted by the calcified lymph nodes (Figures 2B–D). Considering the poor response of the vascular complication to ATT, portal venous angioplasty by balloon dilation and stent implantation was performed via percutaneous transhepatic approach. The proximal-distal pressure gradient of the portal vein decreased from 19 to 6 mmHg. Besides, portal vein at the hepatic hilum was clearly displayed, and collateral varices could not be seen after the procedure (Figure 3). The ascites resolved promptly and continued ATT were administrated after discharge, with no recurrence of ascites occurred during the six-month follow-up.

## Discussion

Globally, TB remains a major public health problem up to now. Though pulmonary TB is the most common type, 16% of TB associated infection occurs in other sites, including abdominal cavity (6). After the establishment of primary infection in the lung, *M. tuberculosis* can disseminate to peritoneal cavity as a result of lymphohematogenous or hematogenous spread from a pulmonary focus. Also, it can pass through the intestinal mucosa to the mesenteric lymph nodes after swallowing bacilli (7). Peritoneum and lymph nodes are the most common sites of abdominal TB (8, 9). Generally, tuberculous peritonitis can be divided into wet ascitic type with massive free or located ascites, dry plastic type with caseous nodules and fibrosed peritoneum, and fibrotic fixed type with masses involving the mesentery, omentum and peritoneum (3). The intra-abdominal infection could cause high frequency of portal vein thrombosis (PVT) or even CTPV, contributing to an increase in portal venous pressure. Organs and lymph nodes compression are main extravascular mechanisms of portal venous stenosis caused by abdominal TB. Lymph nodes following *M. tuberculosis* infection could form classical caseous granulomas. Both suppuration due to delay in the treatment and successful treatment of lymph nodes TB could cause nodal calcification (9). Vascular compression and constriction due to lymphadenopathy with calcification, as shown in our case, resulting in prehepatic PHT.

Portal venous obstruction or stenosis is a major cause of non-cirrhotic PHT, but is rarely caused by *M. tuberculosis* infection. By reviewing the literature, we summarized 9 cases of PHT caused by abdominal TB over the past 30 years (Table 1) (10–17). Portal venous stenosis results in increased portal venous pressure, causing further reduction in liver blood perfusion, which may cause deterioration in liver function. Once PHT arises, variceal bleeding and ascites as its frequent complications may occur and composition analysis is crucial for the differential diagnosis of ascites (18). As in current case, SAAG in the early course was <11 g/L, corresponding with tuberculous peritonitis. After receiving standard ATT, systemic manifestations and pleural effusion disappeared whereas ascites recurred with SAAG > 11 g/L, indicating PHT-induced ascites. Another symptom should be differential diagnosed is pancytopenia, which may result from hypersplenism due to PHT, activation of TB or side effects of ATT (19). Thus, once pancytopenia develops in patients receiving ATT, hypersplenism should not be ignored apart from considering the bone marrow suppression of anti-TB agents.

**Table 1:** The clinical characteristics of patients with portal venous obstruction/stenosis caused by abdominal tuberculosis.

**References**	**Gender/age**	**Etiology**	**Manifestation**	**Improvement after ATT**
Ruttenberg et al. (10)	M/26	LN compression	GI bleeding	No
Ruttenberg et al. (10)	F/42	PVT	Esophageal varices, abnormal LFT	NA
Schneider et al. (11)	F/70	PVT	Ascites, hypersplenism	No
Jazet et al. (12)	M/37	LN compression	GI bleeding, abnormal LFT	NA
Liew et al. (13)	M/26	Organ compression, PVT	GI bleeding, abnormal LFT	No
Mojtahedzadeh et al. (14)	F/16	Organ compression	GI bleeding, abnormal LFT	Yes
Ozseker et al. (15)	M/43	PVT	GI bleeding, ascites	Yes
Mohite et al. (16)	F/16	PVT	GI bleeding, abnormal LFT	NA
Díaz Fontenla et al. (17)	M/27	CTPV	Esophageal varices, ascites, hypersplenism	No

*ATT, anti-tuberculosis therapy; LN compression, lymph nodes compression; GI bleeding, gastrointestinal bleeding; PVT, portal venous thrombosis; LFT, liver function test; CTPV, cavernous transformation of portal vein*.

It is a great challenge in the diagnosis of abdominal TB for its variable non-specific presentations and paucibacillary of the lesions. Indirect immunologic tests including purified protein derivative, interferon-γ release assays and serum antibody profiles provide diagnostic evidence of TB but cannot distinguish between latent and active infection (20). Detection of caseating granuloma via biopsy is typical histological features while acid-fast bacilli and TB-DNA test are more specific (21). If ascites exists, SAAG <11 g/L and elevated ADA provide more evidence of TB infection, otherwise, PHT should be taken into consideration. For portal venous stenosis, radiological confirmation is possible using CTA with three-dimensional reconstruction. The portal venous phase of angiography is especially crucial for visualization of the entire portal venous system, including the extent of stenosis and other vascular complications like PVT, CTPV and collateral veins, which are vital for therapeutic decision-making. During the vascular interventional procedure, portal venography can further reveal details of portal vein along with collateral circulation. Portal pressure gradient and hepatic venous pressure gradient measurement can be performed for differential diagnosis between prehepatic and sinusoidal PHT (22).

Standard ATT have limited therapeutic effects on relieving the vascular complications. In cases reported previously (Table 1), only two receiving ATT completely recovered from the stenosis of portal vein without any other treatment aming at PHT (14, 15). One patient underwent surgery to reveal PVT (11) and other two needed prophylactic treatment of variceal bleeding (10, 12). Herein, we described a case of abdominal TB with symptomatic PHT after ATT, suggesting that approaches aiming at relieving portal venous stenosis should be highly considered when lesions response to ATT poorly. Surgical approaches such as thrombectomy or portosystemic shut surgery have been used in portal venous obstruction and Mesoan-Rex shunt (mesenterico-left portal vein bypass) further protects liver from ischemia as a relatively physiologic manner (23). However, technical difficulties, post-operative complications like intra-abdominal adhesion have restricted their clinical application (24). Portal vein angioplasty with or without stent placement as an alternative to surgical shut have been used in various kinds of portal venous stenosis including inflammation, hepatic transplantation, and malignant tumor (25, 26). It has been confirmed of its efficacy and safety with the advantages of higher successful rate, minimal trauma and restoring the physiological hepatic blood flow. Transjugular and ultrasound-guided percutaneous transplenic/transhepatic approach can be chosen and the later was shown to be with fewer side effects (27). On current case, we got recanalization of portal vein by balloon dilation and stent implantation. It restored normal hepatic circulation which directly reduced the portal venous pressure without the cost of reducing blood supply of liver. To our knowledge, it is the first report that application of interventional strategy on portal venous stenosis caused by abdominal TB.

## Conclusion

PHT due to portal venous stenosis is an uncommon vascular complication after abdominal TB infection, and represents a diagnostic and therapeutic challenge. The complications (ascites, pancytopenia) due to PHT should be differentiated from TB reactivation and medication side effects during ATT. CTA and portal venography would provide valuable evidence for the early diagnosis and clinical decision-making of PHT. Portal vein angioplasty with stent placement is a safe and effective treatment to relieve vascular compression, restoring physical structure, and preventing complications of PHT. We suggest that interventional radiological procedure should be considered when conventional anti-TB drugs have failed.

## Data Availability Statement

The raw data supporting the conclusions of this article will be made available by the authors, without undue reservation.

## Ethics Statement

Ethical review and approval was not required for the study on human participants in accordance with the local legislation and institutional requirements. The patients/participants provided their written informed consent to participate in this study.

## Author Contributions

HW: conceptualization and supervision. XQ, YT, and ZW: data curation. YT and HW: funding acquisition and writing—review & editing. HT, BW, and YY: investigation. XQ and YT: writing—original draft. All authors contributed to the article and approved the submitted version.

## Funding

This study was supported by the National Natural Science Fund of China (Grant No. 82000574), Sichuan Science and Technology Program (Grant No. 2020YJ0084), 1·3·5 project for disciplines of excellence - Clinical Research Incubation Project (Grant No. 2019HXFH024), Post-Doctor Research Project (Grant No. 2019HXBH074), and West China Hospital, Sichuan University.

## Conflict of Interest

The authors declare that the research was conducted in the absence of any commercial or financial relationships that could be construed as a potential conflict of interest.

## Publisher's Note

All claims expressed in this article are solely those of the authors and do not necessarily represent those of their affiliated organizations, or those of the publisher, the editors and the reviewers. Any product that may be evaluated in this article, or claim that may be made by its manufacturer, is not guaranteed or endorsed by the publisher.

## Abbreviations

ADA: adenosine deaminase
ATT: anti-tuberculosis therapy
CRP: C-reactive protein
CT: computed tomography
CTA: CT angiography
CTPV: cavernous transformation of portal vein
ESR: erythrocyte sedimentation rate
PHT: portal hypertension
PVT: portal vein thrombosis
SAAG: serum-ascites albumin gradient
TB: tuberculosis
*M. tuberculosis*: *Mycobacterium tuberculosis*.
